# Implementing an Antimicrobial Stewardship Lecture Series for Family Medicine Residency Programs in South Carolina

**DOI:** 10.1017/ash.2024.105

**Published:** 2024-09-16

**Authors:** Kayla Antosz, Pamela Bailey, Majdi Al-Hasan, Brandon Bookstaver, Hana Winders, Sarah Battle

**Affiliations:** ASC-SC, University of South Carolina College of Pharmacy; Prisma Health Midlands/University of South Carolina; University of South Carolina School of Medicine; Prisma Health-Midlands

## Abstract

**Background:** Family medicine physicians are one of the leading prescribers of antimicrobials in both the inpatient and ambulatory setting, however appropriate education on antimicrobial stewardship (AS) is lacking. The Antimicrobial Stewardship Collaborative of South Carolina (ASC-SC) created a family medicine AS lecture series to increase awareness of stewardship, improve antimicrobial prescribing throughout the state, and ultimately combat antimicrobial resistance. **Methods:** All family medicine residency programs in South Carolina (n=17) were contacted to determine interest in a four 1-hour long lecture series regarding various AS topics provided by infectious diseases physicians and pharmacists. The introductory AS lecture included topics such as interpreting minimal inhibitory concentrations, utilizing antibiograms, guidelines for diagnosing and treating common infections, and antibiotic essentials. Lectures were given on-site, and eight identical pre- and post-lecture questions were asked to assess baseline knowledge and efficacy of the introductory lecture. Not all the attendees answered all the pre- and post-lecture questions. A Chi-square analysis was used to determine statistical significance. **Results:** To date, 7 family medicine residency programs were given the introductory antimicrobial stewardship lecture and were included in the total analysis. Respondents included 1st year (25 of 99 responses, 25%) and 2nd year family medicine residents (17 of 99 responses 17%). When asked “How familiar are you with the concept of antimicrobial stewardship?”, 43 of 106 (41%) respondents were at least familiar or very familiar prior to the lecture compared to 81 of 93 (87%) after the lecture (p < 0.001). When asked “How confident are you in using antibiograms for antimicrobial decisions?”, 41 of 107 (38%) were confident or very confident pre-lecture and 83 of 101 (82%) post-lecture (p < 0.001). When given a case-based question on using an antibiogram to determine an appropriate empiric agent for inpatient pyelonephritis, 59 of 107 (55%) respondents were able to answer the question correctly pre-lecture compared to 85 of 99 (86%) post-lecture (p < 0.001). Among those who answered the question incorrectly, 60% selected the agent with the highest percentage susceptible rate in the antibiogram, despite it being an inappropriate agent for pyelonephritis. **Conclusion:** The ASC-SC lecture series was an effective tool to increase awareness and knowledge of antimicrobial stewardship to family medicine providers. This lecture series survey data helps determine what family medicine residents commonly misunderstood in AS concepts and helps guide future initiatives.

